# Sustainable utilization of *Gyrinops walla* Gaetner: in vitro production of sesquiterpenes by chemical and biological elicitation

**DOI:** 10.1186/s43141-021-00187-2

**Published:** 2021-09-03

**Authors:** Sachithri Munasinghe, Seneviratnege Somaratne, Shyama Weerakoon, Chandani Ranasinghe

**Affiliations:** 1 Department of Botany, The Open University of Sri Lanka, Nawala, Sri Lanka; 2 Department of Chemistry, The Open University of Sri Lanka, Nawala, Sri Lanka

**Keywords:** Sesquiterpenes, Sustainability, *Gyrinops walla*, In vitro production, Artificial elicitation

## Abstract

**Background:**

The recent recovery of *Gyrinops walla* as a potential producer of market-quality agarwood in mature damaged woods and branches has led to the intense illicit felling and exportation of *G. walla* leading to the verge of extinction from Sri Lankan flora. The sustainable utilization of *G. walla* undoubtedly enhances the foreign exchange of the country and the non-destructive utilization through tissue culture–based techniques is the only option available for sustainable exploitation and conservation of the vulnerable species. Healthy calli and cell suspensions were chemically and biologically elicited with salicylic acid (SA) and methyl jasmonate (MJ), and the sterilized fungal homogenate (carbohydrate equivalents) of *Fusariym oxysporum*, *Phaeocremonium parasitica*, *Aspergillus niger*, *Trichoderma viride*, *Penicillium commune* and *Lasidiplodia theobromae* fungal strains, respectively. The elicited calli and cell suspensions were harvested at different time periods to extract sesquiterpenes.

**Results:**

Sesquiterpenes were produced in calli under chemical elicitors with media concentrations of 10 μM SA, 100 μM SA, 10 mM MJ and 1 mM MJ and cell suspensions under 0.5 μM SA and 0.1 mM MJ. *Phaeocremonium parasitica*, *Trichoderma viride* and *Lasidiplodia theobromae* were more effective in the production of sesquiterpenes in *G. walla* callus and cell suspension by biological elicitation.

**Conclusion:**

The findings of the study led to the conclusion of the possibility of induction of production of sesquiterpenes through elicitation of *G. walla* calli and cell suspension in an in vitro system for sustainable utilization and conservation endeavours.

## Background

*Gyrinops* is a member of the Thymelaeaceae family [1], and similar to *Aquilaria*, *Gyrinops* is also classified as agarwood-producing trees [2]. Agarwood is a dark resinous substance, extremely appreciated in luxury perfumery and medicines. As a response to the stress of wounding and microbial infection, agarwood-producing species may activate their plant defence system triggering the production of agarwood that aids in suppressing and localizing the infected area which helps the plant to resist or delay the infection by tylosis [3]. Fragrant compounds of agarwood are known to be sesquiterpenoids and chromone derivatives, which are the main source of agarwood’s characteristic odour [4].

The products from naturally formed agarwood demand high economic value in the world fragrance market and the supply of agarwood from Southeast Asian countries is decreasing, agarwood from *G. walla* in Sri Lanka has drawn boundless attention from high-end users and traders. As a result, within 2013, the police and customs of Sri Lanka had confiscated 13,800 kg of processed agarwood chips [5] (www.dailynews.lk) and from 2015 to 2016, about 89,212 kg of agarwood chips has been confiscated when trying to export illegally for countries India, South Africa and United Arab Emirates [6]. In December 2012, the Biodiversity Secretariat of Sri Lanka re-categorized *G. walla* as “vulnerable” species according to IUCN Red List Categories [7] after which the export of timber, tissues or any extracts from the species was banned. Under these circumstances, there is a need for the conservation of this valuable species while avoiding artificial induction of agarwood which involves destructive harvesting and maintaining profitable export of agarwood from the country. Therefore, the application of plant biotechnological methods for sustainable utilization of *Gyrinops walla* is evident.

Callus and cell suspension culture systems are used nowadays for the large-scale production of plant cells from which secondary metabolites are extracted. The major advantage of cell culture is that synthesis of bioactive secondary metabolites is possible running in a controlled environment, independently from climate and soil conditions [8]. Cell cultures assist in two major ways in the production of plant secondary metabolites by yielding defined standard phytochemicals in large volumes and eliminating the presence of interfering compounds that occur in field-grown plants [9]. A few medicinally important alkaloids, anticancer drugs, recombinant proteins and food additives are produced in various cultures of plant cells and tissues in bioreactors. Developments made in the callus and cell culture have made the possibility of producing a wide range of medicinally important compounds such as alkaloids, terpenoids, steroids, saponins, phenolics, flavonoids and amino acids [10]. The first attempt to establish plant cell culture techniques for *Aquilaria* species was reported in 2005 by Qi et al. [11] which successfully established cell suspensions from root tissue obtained from in vitro germinated plantlets of *A. sinensis*, while Okudera and Ito [12] established cell suspension cultures using leaf tissue from seedlings of *A. crassna*, which had been germinated and grown in greenhouses. To date, no studies have been reported on the establishment of cell cultures and/or production of secondary metabolites in *Gyrinops walla*. The present study reports the feasibility of in vitro producing sesquiterpenes using chemical and biological methods.

## Methods

### Materials

Based on the procedures followed by Okudera and Ito, [12] and Qi et al., [11], salicylic acid (SA) (Sigma Aldrich, USA) and methyl jasmonate (MJ) (Sigma Aldrich, USA) were chosen as the chemical elicitors.

The sterilized fungal homogenate (carbohydrate equivalents) was used as the elicitor. Pure cultures of the fungal strains of *Fusariym oxysporum* [13], *Phaeocremonium parasitica* [14], *Aspergillus niger* [15], *Trichoderma viride* [16], *Penicillium commune* [15] and *Lasidiplodia thoebromae* [15] were obtained from the Botany Laboratory, The Open University of Sri Lanka (OUSL), Nawala, Sri Lanka.

### Establishment of calli and cell suspensions

Calli were raised according to Munasinghe et al. [17] and cell suspensions were initiated by placing 2 g of leaf-derived callus in a 50-mL conical flask containing 20 mL of liquid culture medium (MSM + 2.5 mg/L NAA + 0.5 mg/l BAP), without gelling agent and charcoal. It was placed on a shaker incubator (75–130 rpm) in continuous darkness and the total volume of liquid medium was gradually increased to 50 mL over a period of 3 weeks [12]. After 3 weeks, cell viability was tested using the 2, 3, 5-triphenyltetrazolium chloride (TTC) reduction method [18]. Once cell suspensions were established, they were sub-cultured at 3-week intervals in which 5 mL of cells (SCV — settled cell volume) was transferred into 100-mL conical flasks containing 50 mL of liquid MSM. During each sub-culturing, large cell clumps were manually removed using sterile forceps and continuously sub-cultured until a uniform cell suspension was established. Once a uniform cell suspension line has been established, cell viability was tested using TTC [19].

### Chemical elicitation

#### Exposing callus culture to chemical elicitors

The experiment was carried out in a Petri plate containing MS medium and each flask was started with about 2 g of fresh weight calli as the inoculum. SA was added to the MS medium supplemented with 30.0 g/L sucrose, 100.0 mg/L myo-inositol, 10 g/L agar, 0.5 mg/L BAP and 2.5 mg/L NAA with a final concentration of 10μM (1.38 mg/L) and 100μM (13.8 mg/L), separately [12]. Based on [11], same procedure was repeated with MJ with a final concentration of 0.1mM (0.24g/L), 10mM (2.24g/L), 100mM (22.4 g/L) and 1M (224.3 g/L), separately. The calli were incubated at 25 °C in the dark for 2 to 16 weeks and harvested every 2 weeks for analysis by GC-MS. Visual observations were based on medium colour and sensory evaluation was by sniffing the flask immediately after incubation. All experiments were conducted in triplicate. Cell viability was accessed using the 2, 3, 5-triphenyltetrazolium chloride (TTC) reduction method during the incubation period.

#### Exposing cell culture to chemical elicitors

The experiment was carried out in a 100-mL Erlenmeyer flask containing 50 mL of fresh MS medium supplemented with 30.0 g/L sucrose, 100.0 mg/L myo-inositol, 0.5 mg/L BAP and 2.5 mg/L NAA. Each flask was started with ca. 1 g of fresh weight 3-week-old cell suspension as the inoculum. The cells were grown in the dark on a rotary shaker at 100 rpm for 10 days before treatments and the flasks with 10-day-old cell suspension were exposed to MJ by adding the concentrations of 0.05 mM (11.2 mg/L), 0.1mM (22.4 mg/L) and 0.15 mM (33.6 mg/L) to the medium, separately [11, 20]. Based on Okudera and Ito [12], the same procedure was repeated with SA with a final concentration of 0.25 μM (34.2 mg/L), 0.5 μM (69 mg/L) and 1 μM (138 mg/L), separately. The cultures were maintained in an orbital shaker at 25 °C in the dark. Cells were harvested on different time durations (0 h, 0.5 h, 2 h, 4 h, 6 h, 12 h, 24 h, and 72 h). Visual observations were made on medium colour and sensory evaluation was also performed by sniffing the flask immediately after incubation. All experiments were conducted in triplicate. Cell viability was assessed using the 2, 3, 5-triphenyltetrazolium chloride (TTC) reduction method during the incubation period.

### Biological elicitation

#### Preparation of biological elicitors

Biological elicitors were prepared according to the method described by Qi et al. [11]. All fungal genera were cultured on potato dextrose agar (PDA) (Oxoid, UK) and incubated at room temperature for 7 days. A 1-cm^2^ area of mycelium was cut and transferred into a 250-mL Erlenmeyer flask containing 100 mL of potato dextrose broth (Oxoid, UK) and incubated at room temperature at 100 rpm shaking incubator for 3 weeks. Fully grown mycelia were collected by filtration using a Whatman filter paper (Grade 1) and washed twice in sterile distilled water. Mycelia were dried at 50 °C overnight followed by homogenization in liquid nitrogen using a mortar and pestle. The mixture was then dissolved in 100 mL of distilled water and left for 24 h at room temperature. Subsequently, it was dried in a rotary evaporator (Heidolph, UK) at 50 °C and autoclaved for 20 min at 121 °C. The autoclaved crude mycelial extract was used as the elicitor. The elicitor dose was measured by the total carbohydrate content of the fungal homogenate, which was determined by the phenol-sulphuric acid method using glucose as the standard [21].

#### Exposing callus to biological elicitors

The experiment was carried out in a Petri plate containing MS medium and each flask was started with about 2 g of fresh weight calli as the inoculum. Each fungal homogenate with the concentration of 25 mg/L, 50 mg/L, 75 mg/L and 100 mg/L was added, separately to MS medium supplemented with 30.0 g/L sucrose, 100.0 mg/L myo-inositol, agar 10 g/L, 0.5 mg/L BAP and 2.5 mg/L NAA. The calli were incubated at 25°C in the dark for 2 to 16 weeks and were harvested every 2 weeks for analysis by GC-MS. Visual observations were made on colour of the culture medium and sensory evaluation was achieved by sniffing the flask immediately after incubation. All experiments were conducted in triplicate. Cell viability was tested using the 2, 3, 5-triphenyltetrazolium chloride (TTC) reduction method during the incubation period.

#### Exposing cell suspension to biological elicitors

The experiment was carried out in a 100-mL Erlenmeyer flask containing 50 mL of fresh MS medium supplemented with 30.0 g/L sucrose, 100.0 mg/L myo-inositol, 0.5 mg/L BAP and 2.5 mg/L NAA. Each flask was initiated with *ca.* 1 g of fresh weight 3 weeks old cell suspension as the inoculum. The cells were grown in the dark on a rotary shaker at 100 rpm for 10 days before treatments. After 10 days of growth, fungal homogenates were added to the medium to final concentrations of 2, 4, 6, 8, and 10 mg/L, separately. The cultures were maintained in an orbital shaker at 25 °C in dark. Cells were harvested on different time periods (0 h, 0.5 h, 2 h, 4 h, 6 h, 12 h, 24 h, and 72 h) following the treatment with fungal elicitors [16]. Visual observations were made on the colour of the medium and sensory evaluation was by sniffing the flask immediately after incubation. All experiments were conducted in triplicate. Cell viability was assessed using the 2, 3, 5-triphenyltetrazolium chloride (TTC) reduction method during the incubation period.

#### Extraction of agarwood type constituents of elicited calli and cell suspensions

Extraction of secondary metabolites in elicited calli and cell suspensions was carried out according to Jain et al. [22] modified with sonification. Harvested callus and cell suspension were dried at 50 °C in the oven overnight to a constant weight. The dried calli and cell suspensions were subsequently ground to a fine powder in liquid nitrogen, using a mortar and pestle. Approximately 5 g of the fine powder was transferred into a 50-mL Erlenmeyer flask containing 10 mL of 100% hexane and incubated at room temperature at 150 rpm shaking incubator for 3 days. Subsequently, the sample was sonicated using an ultrasonic water bath for 60 min. The sample was centrifuged in 2500*g* for 10 min and the supernatant was transferred to a new vessel. The resultant supernatant was evaporated at room temperature to remove excess solvent and stored in 4 °C until chemicals analysis.

### Chemical analysis

Chemical analyses were performed at the Herbal Division, Industrial Technology Institute (ITI), Malabe. Thin-layer chromatography (TLC) was performed using pre-coated Silica gel 60 GF254 plates and approximately 6 μl of each sample was spotted on the TLC plate, air-dried and placed in the chromatographic chamber previously saturated with the solvent system (25% ethyl acetate and 75% hexane). Developed TLC plates were observed under UV 366 nm, 245 nm and after spraying with vanillin spray reagent followed by heating at 105 °C for 3–5 min.

The GC-MS was run with a Thermo Scientific Trace 1300 (USA) fused with silica HP-5MS capillary column (30 m × 0.25 mm × 0.25μm). The oven temperature was programmed initially at 50 °C for 2 min following 200 °C for 1 min with a rate of 40 °C/min and at 320 °C for 15 min with a rate of 3 °C/min. The gas chromatogram was coupled to an ISQDD mass selective detector (Thermo Scientific, USA). The MS operating parameters were ionization voltage, 70 eV, and ion source temperature, 250 °C. High-purity (over purity 99.99%) helium was used as the carrier gas. Identification of compounds was based on comparisons of their mass spectra with those recorded in the National Institute of Standards and Technology database (NIST, Version 2.09, MD, USA).

### Data analysis

The descriptive statistics such as mean and standard deviation (SD) were calculated for the data obtained. Further, the relationship between the incubation time and area percentages of sesquiterpenes were graphically presented along with the trends. The inferential statistics such ANOVA and Tukey’s honest test were performed to compare the means. All statistical analyses were performed using SAS (Ver. 9) (2008).

## Results

Effectiveness of the production of sesquiterpenes in calli and cell suspensions subjected to different stress stimuli were initially assessed via visual observation on callus/cell suspension colour, sensory evaluation by sniffing before harvesting and TLC profiles observed under UV 366 nm, which had similar profiles to that of hexane extracts *G. walla* wood after harvesting. The results were used to determine the stress conditions to be further analysed via GC-MS to assess the amount of sesquiterpenes. Considering the relative abundance of sesquiterpenes detected via GC-MS, the relative percentage of four (04) prominent sesquiterpenes, namely γ-selinene, β-caryophyllene, α-cadinol and α-guaiene, were used to assess the productivity of each elicitation method and their conditions. Silane and siloxane compounds were neglected in obtained chromatograms.

### Chemical elicitation of *G. walla* callus and cell suspension

The changes in callus were noted during the stress under varying concentrations of chemical elicitors at different time intervals. In the control, apparent changes of the callus were not observed. On chemical elicitation with 10 μM SA and 100 μM SA, spots on TLC appeared on the 8th and 6th week, respectively. Calli turned to brown after the 10th week and 12th week and spots were not observed on the TLC. Chemical elicitation with MJ induced browning in most of the experimented calli and TLC spots were obtained after the 8th week except for MJ 100 mM. During the chemical elicitation of *G. walla* calli, odour was not sensed in both elicitors.

**Fig. 1:**
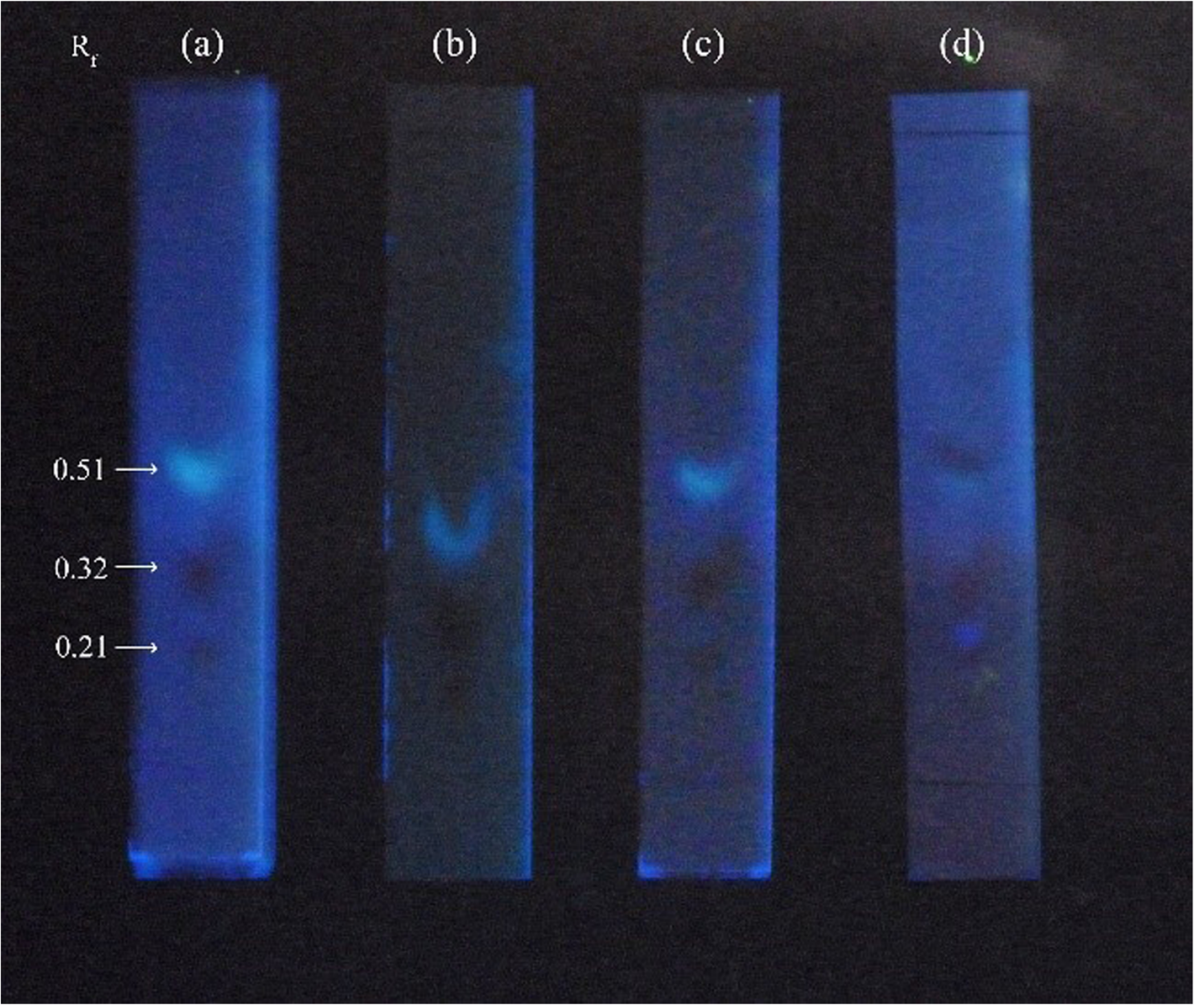
TLC profiles resulting from the chemically elicited calli (**a**) SA 10 μM, (**b**) SA 100 μM, (**c**) MJ 1 mM and (**d**) MJ 10 mM on the 10th week

Three spots with R_f_ values of 0.51, 0.32 and 0.21were observed in TLC profiles of chemically elicited *G. walla* calli. These spots of prominent light blue and maroon in colour were observed in all the TLC profiles of chemically elicited *G. walla* calli (Fig. 1).

**Fig. 2:**
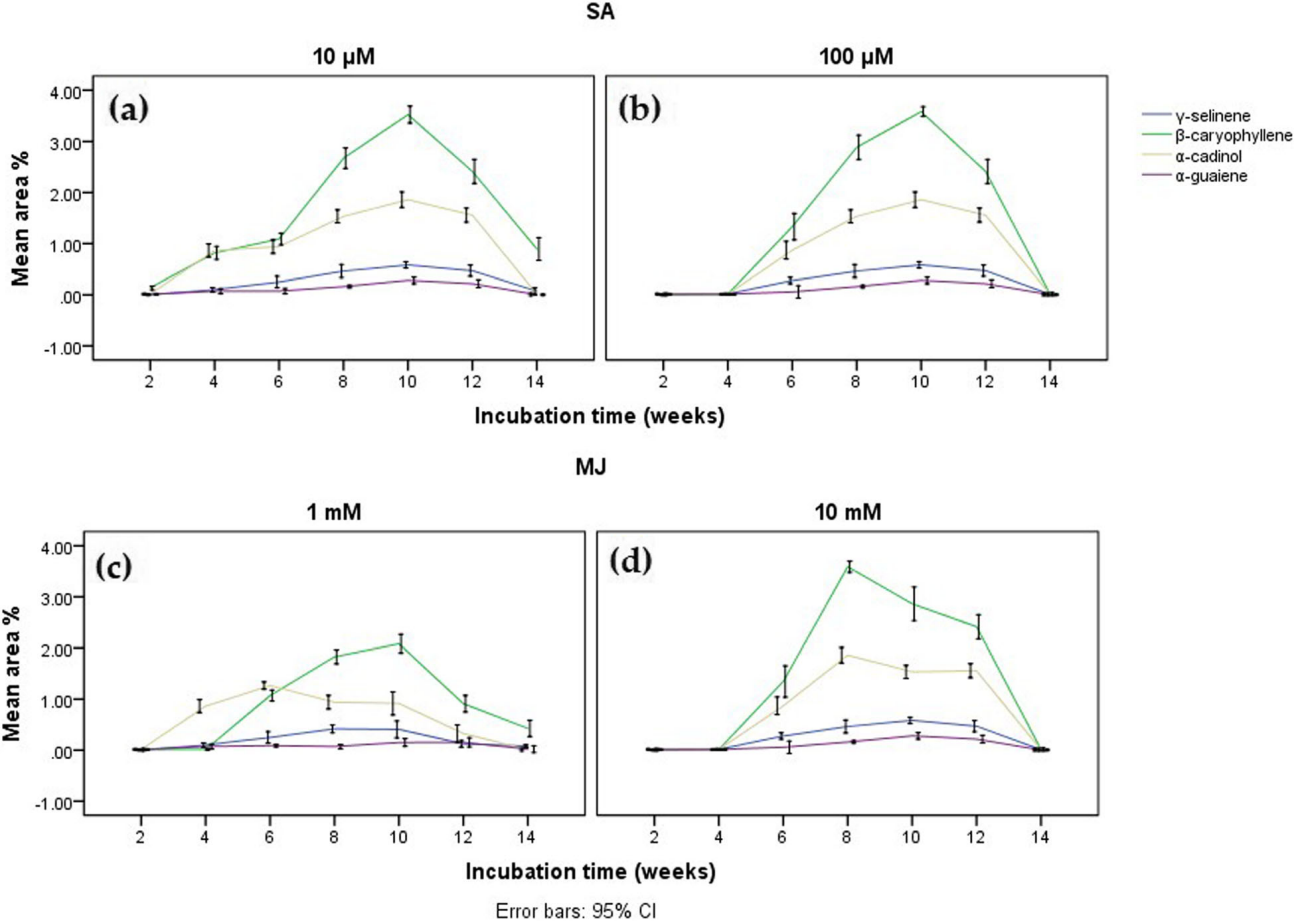
Mean area percentages of sesquiterpenes produced in *G. walla* calli under (**a**) 10 μM SA, (**b**) 100 μM SA, (**c**) 1mM MJ and (**d**) 10mM MJ

**Fig. 3:**
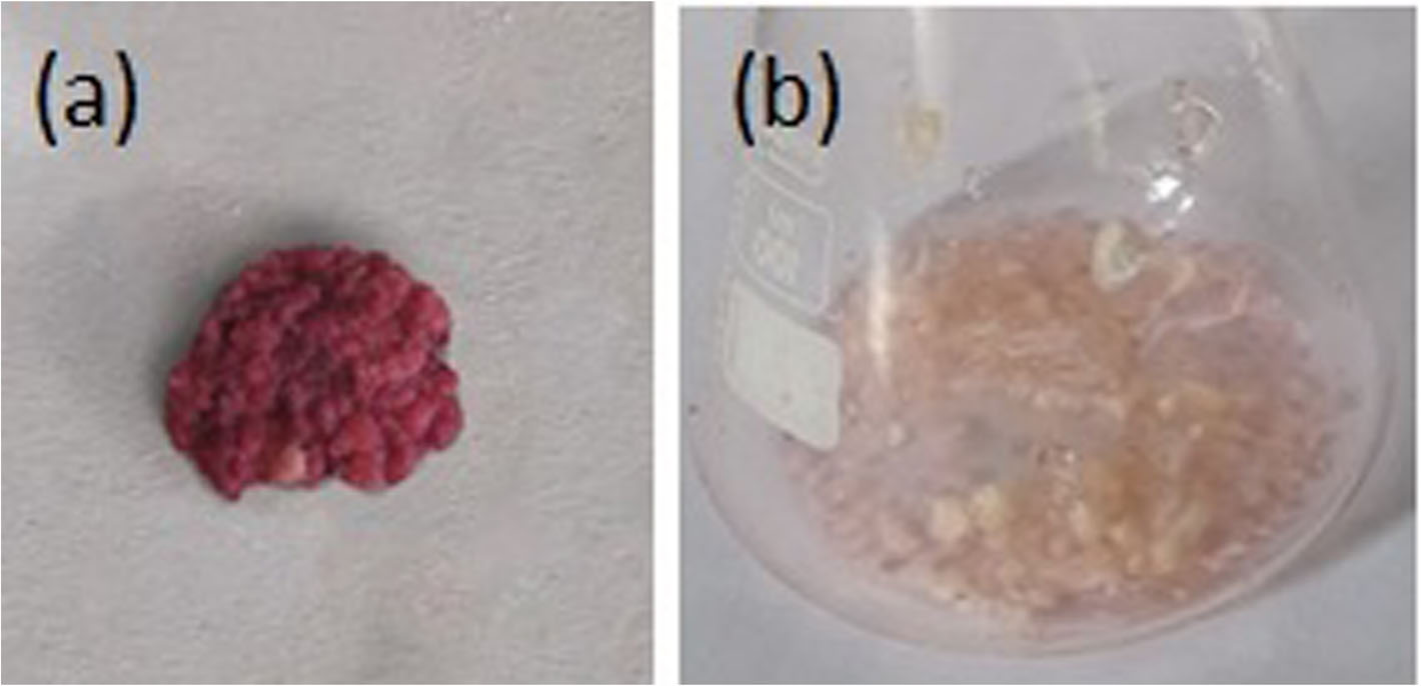
Cell viability of *G. walla* (**a**) calli and (**b**) cell suspension after the TTC reduction test

Results obtained from GC-MS chromatograms of the elicited calli resulted in curve-shaped graphs, indicating the response of calli in the occurrence of sesquiterpenes during the incubation period. The maximum area percentage of desired sesquiterpenes was observed for calli, which were elicited with SA and MJ, on the 8th and 10th week (Fig. 2). Comparatively, the maximum area percentage of sesquiterpenes under the treatment of SA produced sesquiterpenes in 10 μM SA which were equal to that in 100 μM SA (Table 1). On the other hand, the maximum area percentage of sesquiterpenes were obtained in 10 mM of MJ with 3.59% of β-caryophyllene and 1.86% of α-cadinol on the 8th week, and 0.58% of γ-selinene and 0.28% of α-guaiene on the 10th week (Table 1). In comparison, the area percentage of β-caryophyllene is higher in all elicitor conditions while γ-selinene and α-guaiene are the lowest (Fig. 2). In general, the effect of SA and MJ on the area percentage of sesquiterpenes showed a tendency of producing similar results. Cell viability with the TTC reduction test indicated that elicited calli were viable only up to the 8th or 10th week during the incubation period (Fig. 3a).

**Table 1:** Effect of Chemical elicitors on *Gyrinops walla* calli

Type of elicitation	Concentration	Incubation period (weeks)	Area percentage of			
			γ-Selinene	β-Aryophyllene	α-Cardinol	α-Guaiene
Control	0	0	0.00 (0.005)^g^	0.13 (0.01)^i^	0.01 (0.005)^g^	0.01 (0.005)^f^
Salicylic acid	10 μM	2	0.00 (0.005)^g^	0.13 (0.01)^i^	0.01 (0.005)^g^	0.01 (0.005)^f^
Salicylic acid	10 μM	4	0.10 (0.01)^ef^	0.81 (0.05)^gh^	0.86 (0.05)^e^	0.07 (0.02)^d^
Salicylic acid	10 μM	6	0.25 (0.04)^d^	1.09 (0.04)^g^	0.94 (0.05)^de^	0.07 (0.02)^d^
Salicylic acid	10 μM	8	0.46 (0.05)^b^	2.67 (0.08)^b^	1.53 (0.05)^b^	0.16 (0.01)^c^
Salicylic acid	10 μM	10	**0.58 (0.02)**^**a**^	**3.52 (0.06)**^**a**^	**1.86 (0.06)**^**a**^	**0.28 (0.02)**^**a**^
Salicylic acid	10 μM	12	0.47 (0.04)^b^	2.41 (0.09)^c^	1.55 (0.05)^b^	0.21(0.03)^b^
Salicylic acid	10 μM	14	0.07 (0.02)^fg^	0.89 (0.09)^gh^	0.01 (0.01)^g^	0.01 (0.005)^f^
Salicylic acid	100μM	2	0.01 (0.005)^g^	0.01 (0.001)^i^	0.01 (0.003)^g^	0.01 (0.005)^f^
Salicylic acid	100 μM	4	0.01 (0.005)^g^	0.01 (0.005)^i^	0.01 (0.005)^g^	0.01 (0.003)^f^
Salicylic acid	100 μM	6	0.27 (0.02)^d^	1.33 (0.10)^f^	0.87 (0.07)^e^	0.05 (0.01)^de^
Salicylic acid	100 μM	8	0.46 (0.05)^b^	2.88 (0.09)^b^	1.53 (0.05)^b^	0.16 (0.01)^c^
Salicylic acid	100 μM	10	**0.58 (0.02)**^**a**^	**3.58 (0.03)**^**a**^	**1.86 (0.06)**^**a**^	**0.28 (0.02)**^**a**^
Salicylic acid	100 μM	12	0.47 (0.04)^b^	0.24 (0.09)^c^	1.55 (0.05)^b^	0.21 (0.03)^b^
Salicylic acid	100 μM	14	0.01 (0.001)^g^	0.01 (0.001)^i^	0.01 (0.001)^g^	0.01 (0.005)^f^
Methyl jasmonate	1 mM	2	0.01 (0.005)^g^	0.01 (0.001)^i^	0.01 (0.005)^g^	0.01 (0.005)^f^
Methyl jasmonate	1 mM	4	0.10 (0.01)^ef^	0.01 (0.005)^i^	0.86 (0.05)^e^	0.07 (0.02)^d^
Methyl jasmonate	1 mM	6	0.25 (0.04)^d^	1.07 (0.04)^g^	**1.26 (0.02)**^**c**^	0.09 (0.01)^d^
Methyl jasmonate	1 mM	8	**0.41 (0.03)**^**bc**^	1.82 (0.05)^e^	0.94 (0.05)^de^	0.07 (0.01)^d^
Methyl jasmonate	1 mM	10	0.40 (0.06)^c^	**2.08 (0.07)**^**d**^	0.91 (0.09)^d^	**0.15 (0.03)**^**c**^
Methyl jasmonate	1 mM	12	0.12 (0.02)^e^	0.91 (0.06)^gh^	0.32 (0.06)^f^	**0.15 (0.03)**^**c**^
Methyl jasmonate	1 mM	14	0.08 (0.01)^fg^	0.43 (0.06)^h^	0.01 (0.001)^g^	0.02 (0.02)^ef^
Methyl jasmonate	10mM	2	0.01 (0.005)^g^	0.01 (0.01)^i^	0.01 (0.005)^g^	0.01 (0.005)^f^
Methyl jasmonate	10 mM	4	0.01 (0.005)^g^	0.01 (0.005)^i^	0.01 (0.003)^g^	0.01 (0.004)^f^
Methyl jasmonate	10 mM	6	0.27 (0.02)^b^	1.34 (0.12)^f^	0.87 (0.07)^e^	0.05 (0.04)^de^
Methyl jasmonate	10 mM	8	0.46 (0.05)^b^	**3.59 (0.04)**^**a**^	**1.86 (0.06)**^**a**^	0.16 (0.01)^c^
Methyl jasmonate	10 mM	10	**0.50 (0.02)**^**a**^	2.86 (0.13)^b^	1.53 (0.05)^b^	**0.28 (0.02)**^**a**^
Methyl jasmonate	10 mM	12	0.47 (0.04)^b^	2.41 (0.09)^c^	1.55 (0.05)^b^	0.21 (0.03)^b^
Methyl jasmonate	10 mM	14	0.01 (0.01)^g^	0.01 (0.01)^i^	0.01 (0.01)^g^	0.01 (0.005)^f^

The mean values are followed by standard deviation within parentheses. The same letter along the columns indicates no statistically significant difference at p ≤ 0.0

The cell suspension of *G. walla* treated with different chemical stimuli (MJ and SA) resulted in lesser browning and TLC spots appeared only for SA 0.5 μM and MJ 0.1 mM after 4 h and onwards. However, on the 72nd hour, TLC spots were not observed for both chemical elicitors. Most significantly, a slight aroma was sensed when *G. walla* cell suspensions were incubated with 0.1 mM MJ on the 6th hour. Cell viability with the TTC reduction test indicated that elicited cell suspensions were viable only up to the 6th or 12th hour during the incubation period and majority of the cell suspensions were not viable on the 72nd hour (Fig. 3b).

**Fig. 4:**
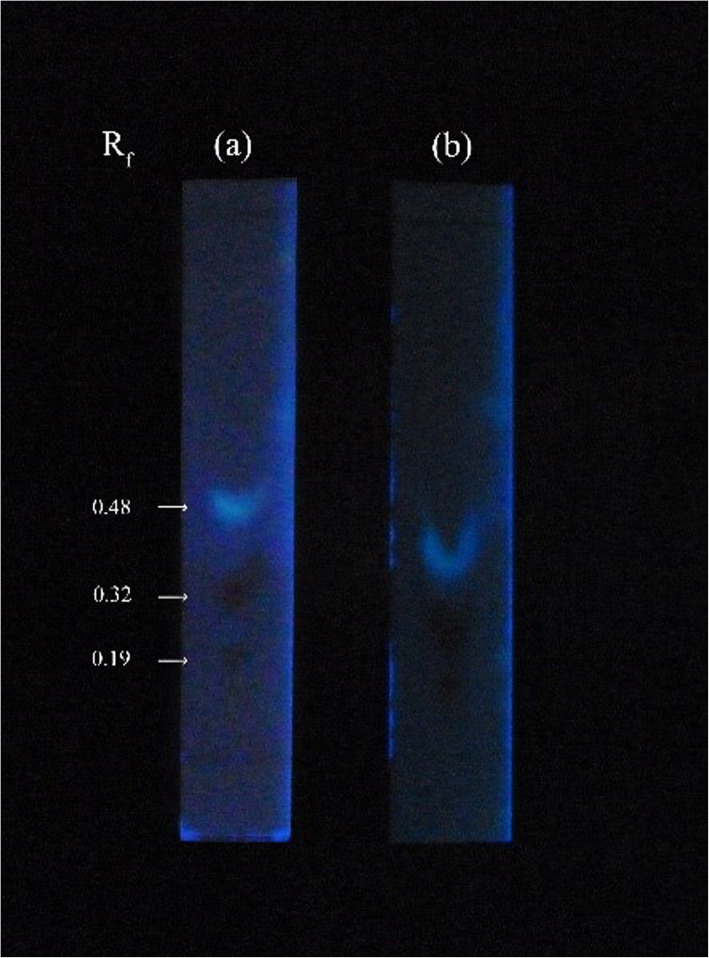
TLC profiles resulting from the chemically elicited cell suspension (**a**) SA 0.5 μM and (**b**) MJ 0.1 mM on the 6th hour

The TLC profiles of *G. walla* cell suspension indicated similar observations to that of *G. walla* calli with prominent light blue and maroon spots (Fig. 4).

**Fig. 5:**
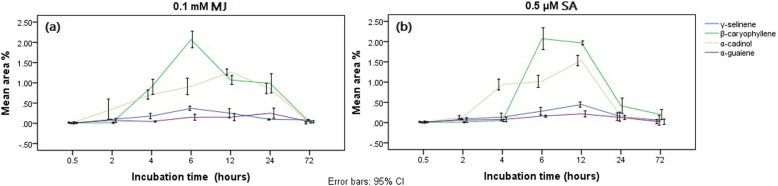
Mean area percentages of sesquiterpenes produced in *G. walla* cell suspension under (**a**) MJ and (**b**) SA

Similar patterns were observed in cell suspensions under SA and MJ with the highest area percentage of sesquiterpenes on the 6th and 12th hour (Fig. 5). A higher area percentage of γ-selinene (0.44%) and α-cadinol (1.53%) was reported for SA while MJ induced a higher production of α-guaiene (0.25%) and β-caryophyllene (2.07%) (Table 2). Earlier production of α-cadinol and later production β-caryophyllene were prominent while cell suspensions were elicited with SA and MJ (Fig. 5). This pattern of chemical appearance was similar to *G. walla* calli with maximum area percentages of β-caryophyllene and α-cadinol, and minimum area percentages of γ-selinene and α-guaiene during the incubation period for both elicitors.

**Table 2:** Effect of Chemical elicitors on *Gyrinops walla* cell suspension

Type of elicitation	Concentration	Incubation period (hours)	Area percentage of			
			γ-Selinene	β-Caryophyllene	α-Cardinol	α-Guaiene
Control	0	0	0.01 (0.005)^e^	0.01 (0.01)^e^	0.01 (0.005)^f^	0.01 (0.005)^d^
Salicylic acid	0.5 μM	0.5	0.01 (0.005)^e^	0.01 (0.01)^e^	0.01 (0.005)^f^	0.01 (0.005)^d^
Salicylic acid	0.5 μM	2	0.08 (0.005)^de^	0.01 (0.005)^e^	0.13 (0.01)^f^	0.06 (0.02)^c^
Salicylic acid	0.5 μM	4	0.13 (0.03)^cd^	0.05 (0.01)^e^	0.94 (0.05)^cd^	0.07 (0.02)^c^
Salicylic acid	0.5 μM	6	0.28 (0.03)^b^	2.07 (0.10)^b^	1.02 (0.06)^c^	0.16 (0.01)^b^
Salicylic acid	0.5 μM	12	**0.44 (0.02)**^**a**^	**1.97 (0.02)**^**a**^	**1.53 (0.05)**^**a**^	**0.21 (0.03)**^**a**^
Salicylic acid	0.5 μM	24	0.14 (0.04)^c^	0.41 (0.07)^cd^	0.14 (0.03)^f^	0.12 (0.02)^bc^
Salicylic acid	0.5 μM	72	0.05 (0.04)^d^	0.20 (0.05)^cd^	0.02 (0.002)^f^	0.02 (0.002)^d^
Methyl jasmonate	0.1 mM	0.5	0.01 (0.005)^e^	0.01 (0.001)^e^	0.01 (0.005)^f^	0.01 (0.005)^d^
Methyl jasmonate	0.1 mM	2	0.09 (0.001)^cd^	0.01 (0.005)^e^	0.35 (0.10)^de^	0.07 (0.002)^c^
Methyl jasmonate	0.1 mM	4	0.18 (0.02)^c^	0.90 (0.07)^d^	0.71 (0.04)^e^	0.04 (0.005)^cd^
Methyl jasmonate	0.1 mM	6	**0.37 (0.02)**^**a**^	**2.07 (0.08)**^**b**^	0.91 (0.08)^cd^	0.15 (0.03)^b^
Methyl jasmonate	0.1 mM	12	0.25 (0.04)^b^	1.07 (0.04)^c^	**1.26 (0.02)**^**b**^	0.15 (0.03)^b^
Methyl jasmonate	0.1 mM	24	0.09 (0.005)^cd^	0.99 (0.30)^d^	0.86 (0.01)^e^	**0.25 (0.02)**^**a**^
Methyl jasmonate	0.1 mM	72	0.08 (0.004)^de^	0.04 (0.01)^e^	0.01 (0.003)^f^	0.04 (0.01)^d^

The mean values are followed by standard deviation within parentheses. The same letter along the columns indicates no statistically significant difference at p ≤ 0.05

**Fig. 6:**
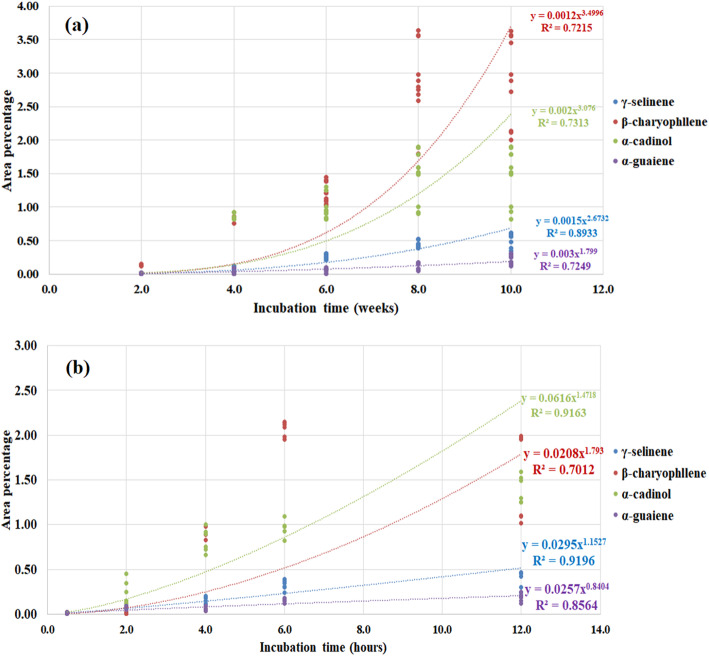
Trends of area percentages of chemically elicited (**a**) *G. walla* calli and (**b**) cell suspension. The graphs present the data up to the maximum area percentage of sesquiterpenes

For *G. walla* calli and cell suspension under the treatment of chemical elicitation, two distinguishable patterns of occurrence of sesquiterpenes were observed (Fig. 6). The area percentage of β-caryophyllene and α-cadinol were higher in both culture conditions while γ-selinene and α-guaiene were lower. Meanwhile, the sesquiterpenes, γ-selinene and α-guaiene in calli and cell suspension behave similarly; β-caryophyllene and α-cadinol act conversely in two culture conditions. Comparatively, earlier production of β-caryophyllene and α-cadinol was observed in *G. walla* calli and cell suspension, respectively.

### Biological elicitation of *G. walla* callus and cell suspension

**Fig. 7:**
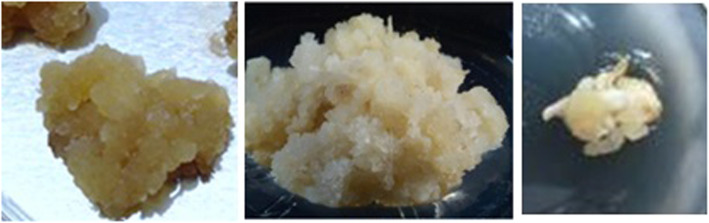
Browning of the calli on elicitation with *L. theobromae*

Six biological elicitors performed differently on each stress condition changing the physical characters of *G. walla* calli. Comparatively, the effect of fungal strains *Fusariym oxysporum*, *Aspergillus niger* and *Penicillium commune* had little or no effect on *G. walla* calli except for browning. In contrast, fungal strains *Phaeocremonium parasitica*, *Trichoderma viride* and *Lasidiplodia theobromae* produced spots on TLC plates on the 4th week onwards and aroma was sensed in some of the elicited conditions. Extensive browning was observed for the elicitation caused by *Lasidiplodia theobromae* that intensified at the end of the incubation period (Fig. 7). Cell viability with the TTC reduction test indicated that elicited calli were viable only up to the 8th or 10th week during the incubation period.

**Fig. 8:**
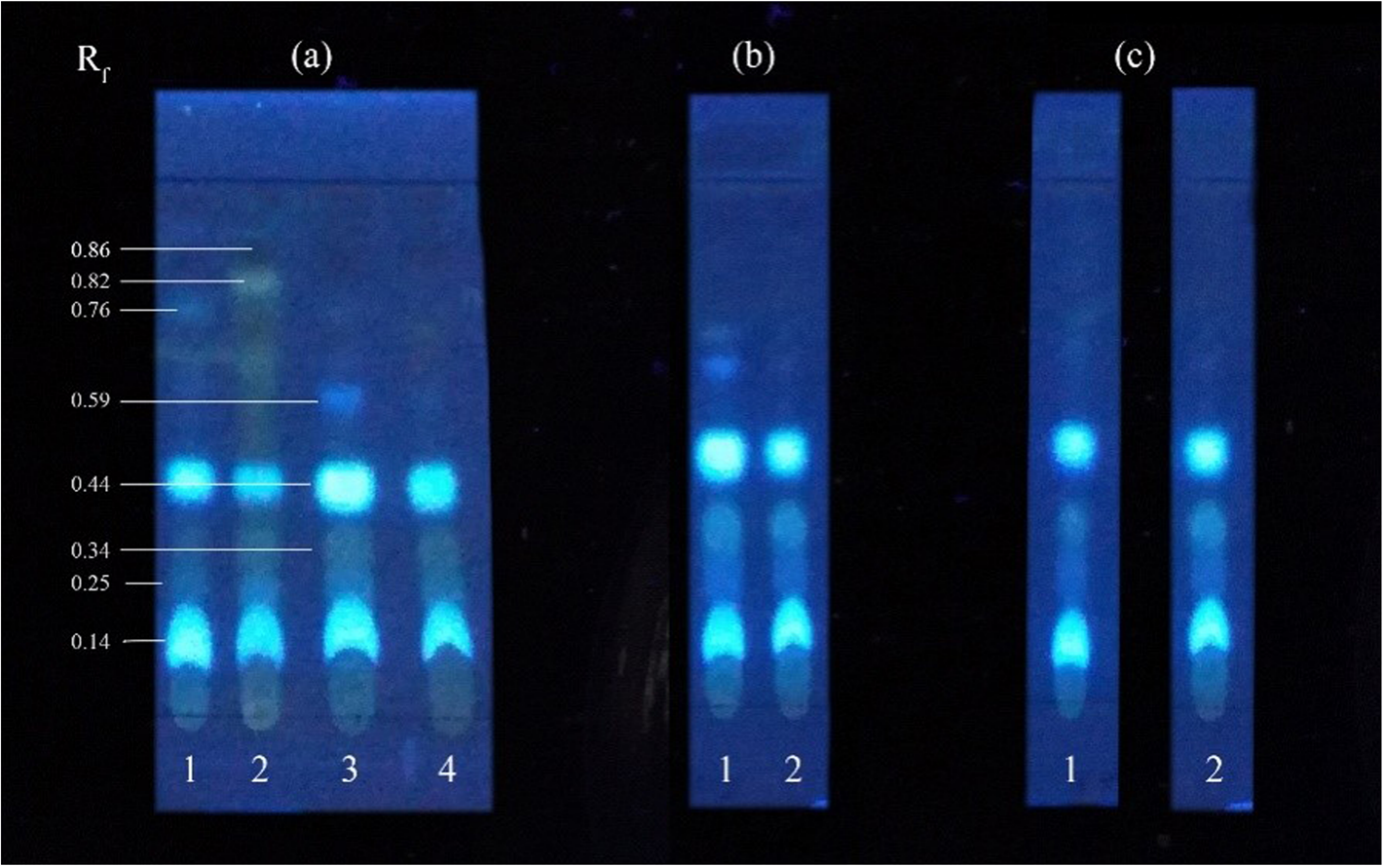
TLC profiles of biologically elicited *G. walla* calli (**a**) *P. parasitica* (1) 25 mg/L, (2) 50 mg/L, 75 mg/L, 100 mg/L; (**b**) *T. viride* (1) 75 mg/L, (2) 100 mg/L; and (***c***) *L. theobromae* (1) 75 mg/L, (2) 100 mg/L on the 10*th* week

TLC profiles of extracts of elicited calli indicted vivid spot patterns with light to dark, blue and yellow in colour with few spots in maroon colour with R_f_ values of 0.86, 0.82, 0.76, 0.59, 0.44, 0.34, 0.25 and 0.14 all three fungal strains: *Phaeocremonium parasitica*, *Trichoderma viride* and *Lasidiplodia theobromae* (Fig. 8).

**Fig. 9:**
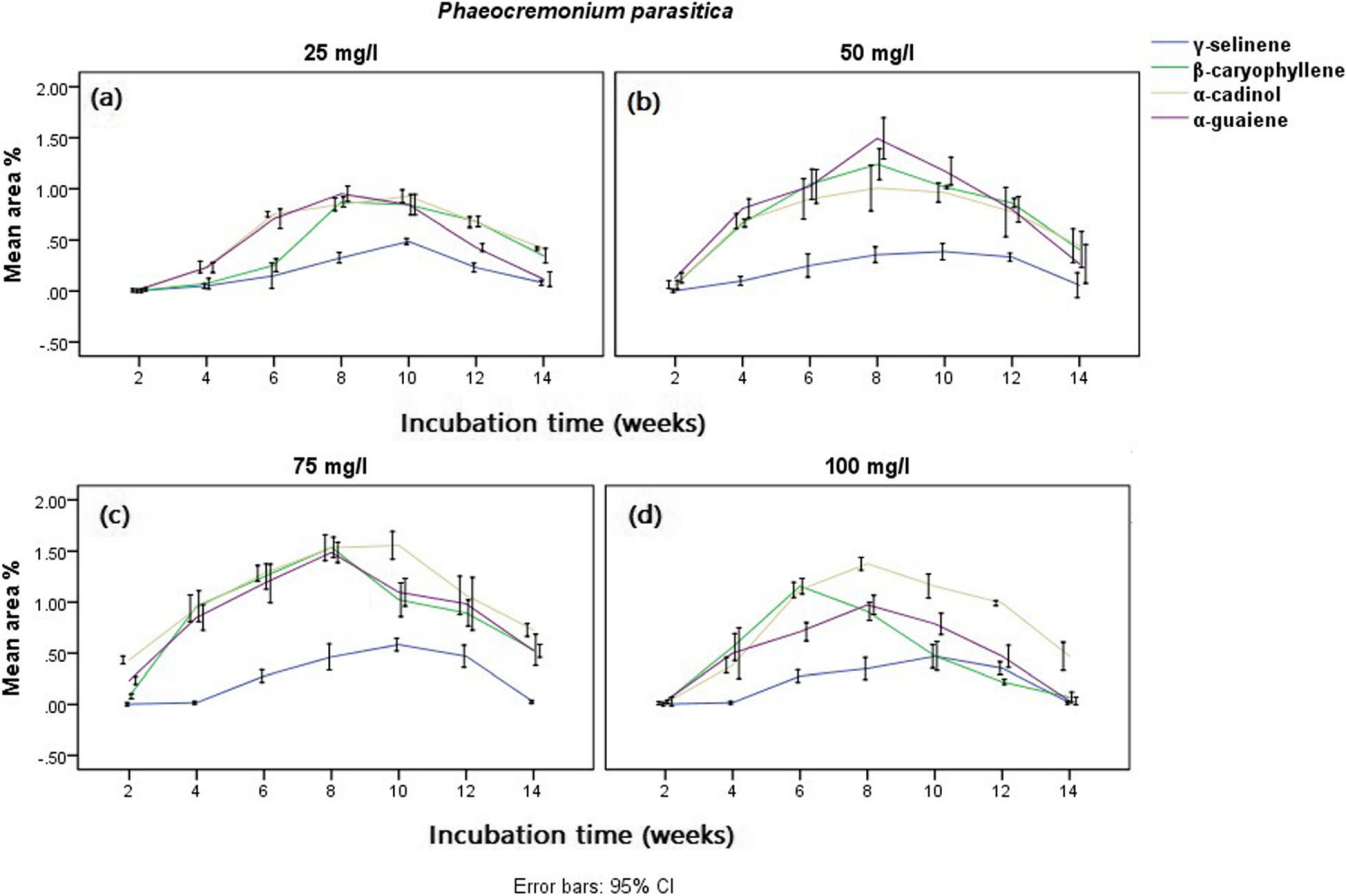
Mean area percentages of sesquiterpenes produced in *G. walla calli* elicited with *P. parasitica* (**a**) 25 mg/l, (**b**) 50 mg/l, (**c**) 75 mg/l and (**d**) 100 mg/l

**Fig. 10:**
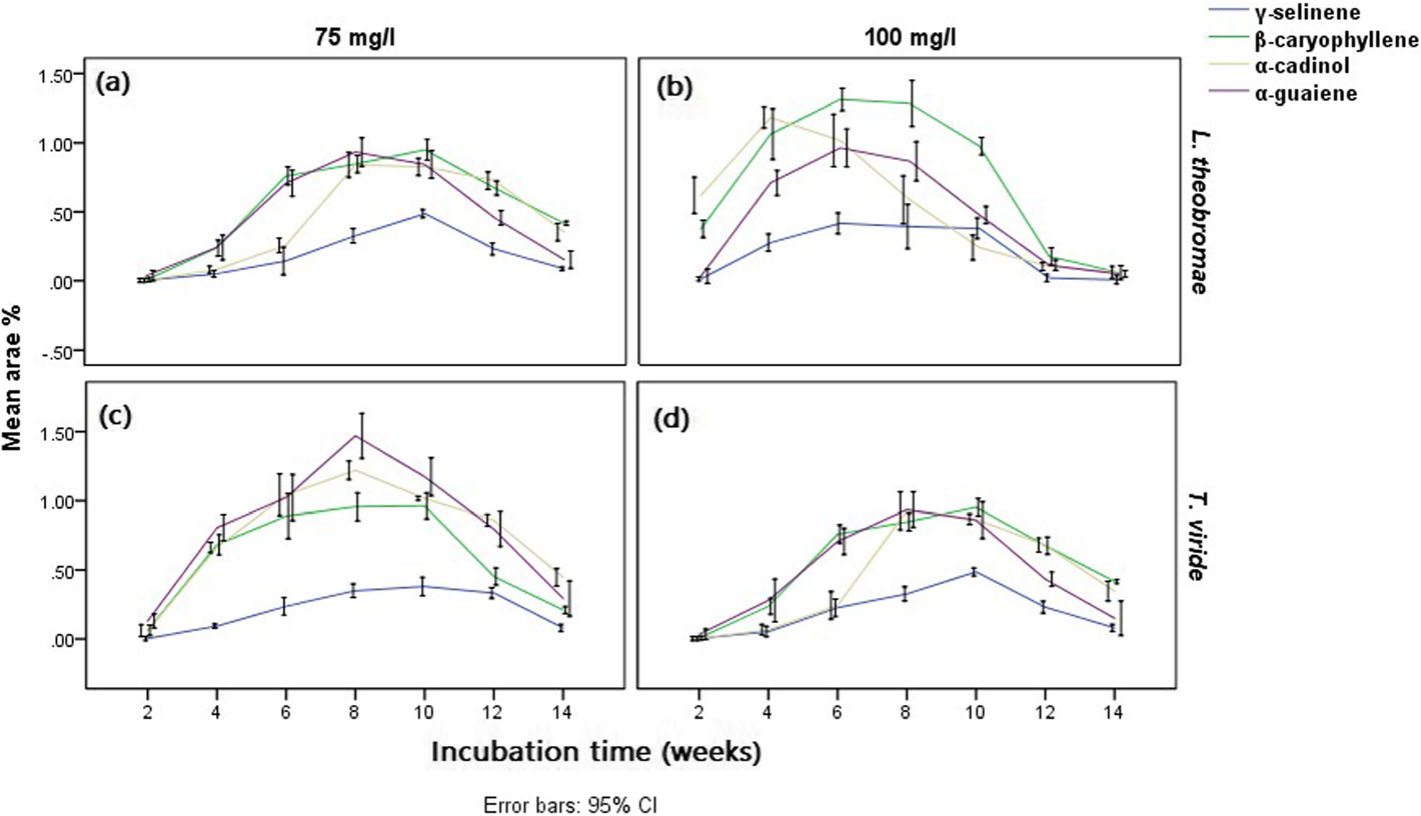
Mean area percentages of sesquiterpenes produced in *G. walla calli* elicited with (**a**) 75 mg/l, (**b**) 100 mg/l *L. theobromae* and (**c**) 75 mg/l, (**d**) 100 mg/l *T. viride*

The results obtained from the GC-MS chromatogram of the elicited *G. walla* calli revealing comparatively a maximum area percentage of sesquiterpenes for three fungal strains are presented graphically (Figs. 9 and 10). The calli elicited by *P. parasitica* indicated a higher area percentage of sesquiterpenes in the 6th, 8th and 10th week of incubation for tested concentrations (Fig. 9). Among the concentrations, the highest area percentage of sesquiterpenes were observed in calli treated with 75 mg/l and kept for 8–10 weeks of incubation (γ-selinene — 0.58%, β-caryophyllene — 1.53% and α-cadinol — 1.55%) (Table 3). Similarly, the concentrations 75 mg/L and 100 mg/L of *Trichoderma viride* were observed to be effective in the biological elicitation of *G. walla* calli, producing the highest sesquiterpene area percentages on the 8th and 10th week (Fig. 10 c and d). Comparatively, a higher area percentage of sesquiterpenes was yielded for calli under 75 mg/L *T.viride* (β-caryophyllene — 0.96%, α-cadinol — 1.22% and α-guaiene — 1.47%)*.* For calli under the concentration of 75 mg/L, *Lasidiplodia theobromae* showed a behavioural pattern similar to that of the other two biological-elicitors, producing a higher area percentage of sesquiterpenes in the 8th and 10th week (Fig. 10 a and b). On the contrary, higher area percentages of desired sesquiterpenes were obtained from the 4th week (α-cadinol) and 6th week (β-caryophyllene, α-guaiene and γ-selinene) under the treatment of 100 mg/L *L. theobromae* (Table 3). Out of the three positive fungal stains and their concentrations, calli incubated under 75 mg/L *P. parasitica* for 8–10 weeks yielded a higher area percentage of three sesquiterpenes comparatively. The area percentages of sesquiterpenes differed from that of chemical and electro-stimulation. Characteristically, higher area percentages of α-cadinol, β-caryophyllene and α-guaiene were observed for three fungal strains which gradually reduced at the end of the incubation period (Figs. 9 and 10).

**Table 3:** Effect of biological elicitors on *Gyrinops walla* calli

Type of elicitation	Concentration (mg/L)	Incubation period (weeks)	Area percentage of			
			γ-Selinene	β-Caryophyllene	α-Cardinol	α-Guaiene
Control	0	0	0.01 (0.005)^n^	0.01 (0.002)^z^	0.01 (0.006)^xy^	0.01 (0.004)^op^
PP	25	2	0.01 (0.005)^n^	0.01 (0.002)^z^	0.01 (0.006)^xy^	0.01 (0.004)^op^
PP	25	4	0.05 (0.002)^mn^	0.07 (0.02)^z^	0.23 (0.02)^wv^	0.23 (0.02)^n^
PP	25	6	0.15 (0.05)^jk^	0.25 (0.02)^xw^	0.75 (0.01)^onp^	0.70 (0.03)^ij^
PP	25	8	0.32 (0.02)^gfh^	**0.87 (0.02)**^**jlimk**^	0.84 (0.02)^oqp^	**0.95 (0.03)**^**feg**^
PP	25	10	**0.48 (0.01)**^**a**^	0.84 (0.04)^lonm^	**0.92 (0.02)**^**jlki**^	0.84 (0.04)^fhg^
PP	25	12	0.23 (0.01)^ji^	0.68 (0.02)^pq^	0.67 (0.05)^rsqp^	0.42 (0.01)^m^
PP	25	14	0.08 (0.01)^mn^	0.34 (0.02)^w^	0.41 (0.005)^u^	0.11 (0.02)^o^
PP	50	2	0.01 (0.0005)^n^	0.06 (0.01)^z^	0.06 (0.01)^xy^	0.13 (0.02)^o^
PP	50	4	0.10 (0.001)^l^	0.66 (0.01)^rq^	0.68 (0.02)^rsq^	0.80 (0.03)^ih^
PP	50	6	0.25 (0.04)^ji^	1.04 (0.06)^fheg^	0.90 (0.08)^jlkm^	1.02 (0.06)^e^
PP	50	8	0.35 (0.03)^efg^	**1.24 (0.06)**^**cd**^	**1.00 (0.09)**^**ghi**^	**1.49 (0.08)**^**c**^
PP	50	10	**0.38 (0.03)**^**ef**^	1.01 (0.005)^fhg^	0.96 (0.03)^jhi^	1.17 (0.05)^d^
PP	50	12	0.33 (0.01)^gfh^	0.86 (0.01)^lnmk^	0.77 (0.09)^onp^	0.79 (0.05)^ij^
PP	50	14	0.05 (0.004)^mn^	0.40 (0.07)^w^	0.44 (0.06)^ut^	0.26 (0.07)^n^
PP	75	2	0.01 (0.005)^n^	0.08 (0.01)^z^	0.43 (0.01)^ut^	0.23 (0.01)^n^
PP	75	4	0.01 (0.005)^n^	0.96 (0.06)^jfhigk^	0.94 (0.05)^jlki^	0.85 (0.05)^fhg^
PP	75	6	0.27 (0.02)^ih^	1.25 (0.05)^c^	1.28 (0.03)^ab^	1.18 (0.07)^d^
PP	75	8	0.46 (0.05)^bcd^	**1.53 (0.04)**^**a**^	1.53 (0.05)^c^	**1.48 (0.04)**^**a**^
PP	75	10	**0.58 (0.02)**^**a**^	1.02 (0.06)^feg^	**1.55 (0.05)**^**a**^	1.09 (0.05)^b^
PP	75	12	0.47 (0.04)^bc^	0.89 (0.05)^ponm^	1.06 (0.07)^gf^	0.98 (0.10)^ef^
PP	75	14	0.02 (0.005)^n^	0.53 (0.06)^st^	0.72 (0.02)^rqp^	0.52 (0.02)^ml^
PP	100	2	0.01 (0.005)^n^	0.03 (0.005)^z^	0.01 (0.005)^xy^	0.02 (0.01)^op^
PP	100	4	0.01 (0.005)^n^	0.56 (0.05)^sr^	0.38 (0.03)^v^	0.50 (0.10)^ml^
PP	100	6	0.27 (0.02)^gih^	**1.15 (0.03)**^**ed**^	1.12 (0.03)^fe^	0.71 (0.03)^ij^
PP	100	8	0.35 (0.04)^efg^	0.91 (0.03)^jfhig^	**1.37 (0.02)**^**b**^	**0.97 (0.03)**^**ef**^
PP	100	10	**0.47 (0.04)**^**bc**^	0.47 (0.05)^stu^	1.15 (0.04)^de^	0.78 (0.04)^ih^
PP	100	12	0.35 (0.02)^ef^	0.22 (0.01)^xw^	0.99 (0.01)^jki^	0.47 (0.04)^ml^
PP	100	14	0.01 (0.005)^mn^	0.08 (0.02)^z^	0.47 (0.01)^ut^	0.03 (0.002)^op^
LT	75	2	0.01 (0.005)^n^	0.01 (0.001)^z^	0.01 (0.004)^xy^	0.03 (0.01)^op^
LT	75	4	0.05 (0.01)^mn^	0.23 (0.02)^wx^	0.08 (0.01)^x^	0.24 (0.03)^n^
LT	75	6	0.14 (0.04)^jk^	0.76 (0.02)^jlmk^	0.25 (0.02)^wv^	0.70 (0.03)^ij^
LT	75	8	0.32 (0.02)^gfh^	0.84 (0.02)^ponq^	**0.84 (0.03)**^**jlkm**^	**0.93 (0.04)**^**feg**^
LT	75	10	**0.48 (0.01)**^**a**^	**0.95 (0.03)**^**jlhigk**^	0.82 (0.02)^onm^	0.84 (0.04)^fhg^
LT	75	12	0.23 (0.01)^ji^	0.67 (0.02)^rq^	0.72 (0.02)^rsqp^	0.45 (0.03)^m^
LT	75	14	0.08 (0.005)^lm^	0.41 (0.005)^w^	0.35 (0.02)^v^	0.15 (0.02)^o^
LT	100	2	0.01 (0.005)^n^	0.37 (0.02)^z^	0.62 (0.05)^s^	0.03 (0.003)^op^
LT	100	4	0.27 (0.02)^ih^	1.06 (0.07)^ef^	**1.18 (0.03)**^**dce**^	0.71 (0.03)^ij^
LT	100	6	**0.41 (0.03)**^**bcde**^	**1.31 (0.03)**^**ab**^	1.01 (0.07)^ghi^	**0.96 (0.05)**^**ef**^
LT	100	8	0.39 (0.06)d^ef^	1.28 (0.06)^bc^	0.58 (0.07)^t^	0.86 (0.05)^fhg^
LT	100	10	0.38 (0.03)^cde^	0.97 (0.02)^jfhig^	0.24 (0.03)^w^	0.47 (0.02)^ml^
LT	100	12	0.02 (0.01)^n^	0.17 (0.02)^lnmk^	0.10 (0.01)^x^	0.11 (0.01)^o^
LT	100	14	0.006 (0.01)^n^	0.06 (0.02)^z^	0.06 (0.01)^xy^	0.05 (0.01)^op^
TV	75	2	0.01 (0.005)^n^	0.06 (0.01)^z^	0.06 (0.01)^xy^	0.13 (0.02)^o^
TV	75	4	0.09 (0.005)^l^	0.68 (0.02)^prq^	0.66 (0.01)^rs^	0.80 (0.03)^ih^
TV	75	6	0.23 (0.02)^ji^	0.89 (0.06)^jlimk^	1.04 (0.06)^gf^	1.02 (0.06)^e^
TV	75	8	0.35 (0.02)^efg^	0.95 (0.04)^fhig^	**1.22 (0.02)**^**cd**^	**1.47 (0.06)**^**c**^
TV	75	10	**0.38 (0.02)**^**ef**^	**0.96 (0.03)**^**jfhigk**^	1.01 (0.005)^ghi^	1.17 (0.05)^d^
TV	75	12	0.33 (0.01)^gfh^	0.45 (0.02)^ponq^	0.86 (0.01)^lnm^	0.79 (0.05)^ih^
TV	75	14	0.08 (0.01)^lmn^	0.21 (0.01)^xw^	0.44 (0.02)^ut^	0.29 (0.05)^n^
TV	100	2	0.01 (0.005)^n^	0.01 (0.004)^z^	0.01 (0.003)^xy^	0.03 (0.01)^op^
TV	100	4	0.05 (0.01)^mn^	0.23 (0.02)^xw^	0.06 (0.01)^xy^	0.28 (0.06)^n^
TV	100	6	0.22 (0.02)^ji^	0.76 (0.02)^jlmk^	0.24 (0.04)^wv^	0.70 (0.03)^ij^
TV	100	8	0.32 (0.02)^gfh^	0.84 (0.02)^ponq^	**0.92 (0.05)**^**jlkm**^	**0.93 (0.05)**^**feg**^
TV	100	10	**0.48 (0.01)**^**a**^	**0.95 (0.02)**^**jlhik**^	0.86 (0.01)^nm^	0.86 (0.05)^fhg^
TV	100	12	0.23 (0.01)^ji^	0.67 (0.02)^prq^	0.68 (0.02)^rsq^	0.43 (0.02)^m^
TV	100	14	0.08 (0.01)^mn^	0.41 (0.005)^w^	0.34 (0.02)^t^	0.15 (0.05)^o^

*PP*, *Phaeocremonium parasitica*; *TV*, *Trichoderma viride*; and *LT*, *Lasidiplodia theobromae*. The mean values are followed by standard deviation within parentheses. The same letter along the columns indicates no statistically significant difference at p ≤ 0.05

A rapid browning of cell suspension cultures with *Phaeocremonium parasitica*, *Trichoderma viride* and *Lasidiplodia theobromae* fungal strains was observed within 24 h subsequently to the biological elicitation. However, such browning was not observed in the control and the fungal strains *Fusariym oxysporum*, *Aspergillus niger* and *Penicillium commune*. Smell was detected in higher concentrations of biological elicitors (8 mg/L and 10 mg/L) of *Phaeocremonium parasitica* and *Lasidiplodia theobromae* after 6 h of incubation and *Trichoderma viride* indicated no sign of change in the cell suspension. Cell viability with TTC reduction test indicated that elicited cell suspensions were viable only upto 6th or 12th hour during the incubation period and majority the cell suspensions were not viable in the 72nd hour. However, the TTC reduction test on *Trichoderma viride* indicated that cell suspensions in all the incubation periods were not viable.

**Fig. 11:**
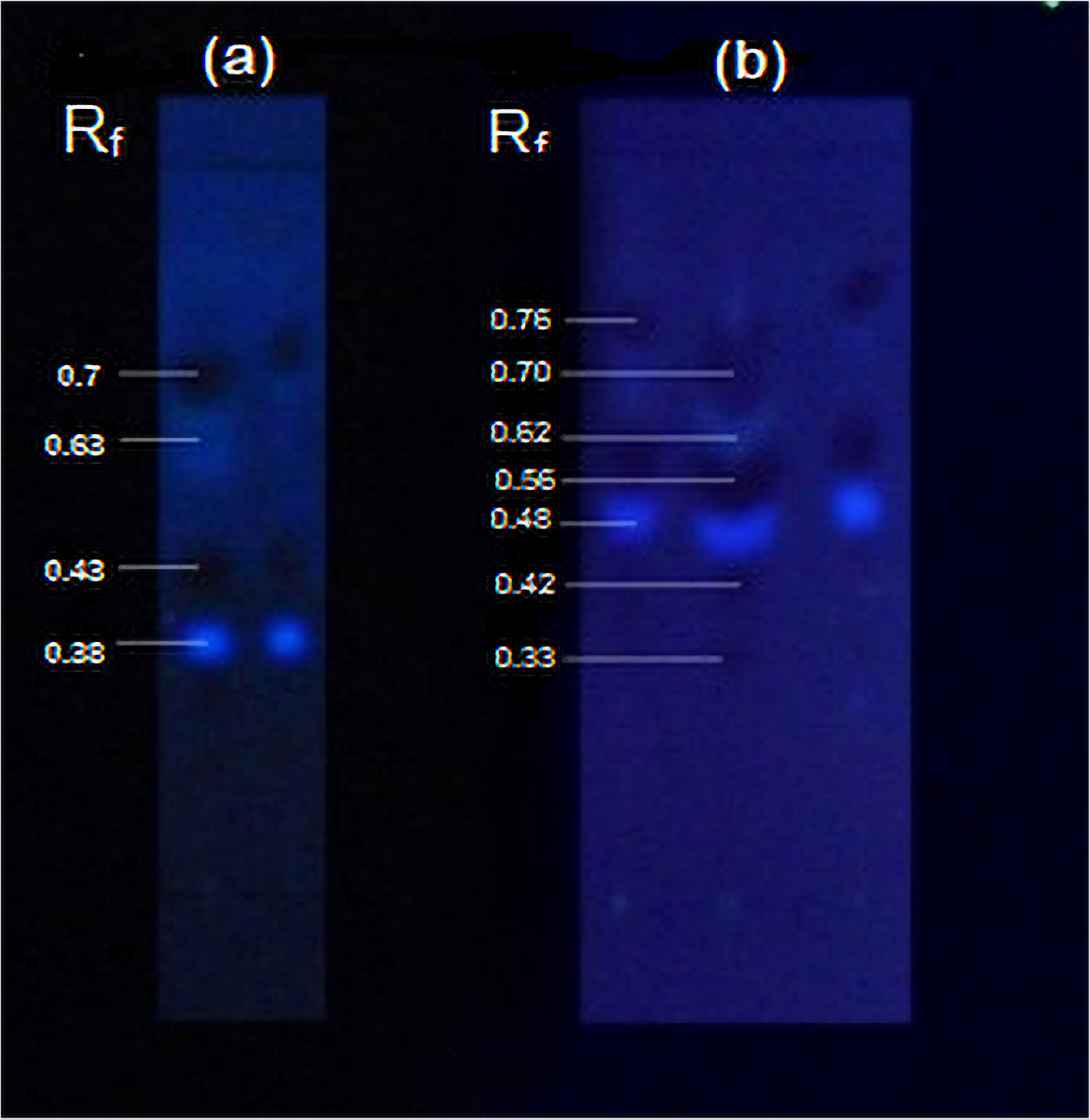
TLC profiles of biologically elicited *G. walla* cell suspension (**a**) *L. theobromae* (1) 8 mg/L, (2) 10 mg/L and (**b**) *P. parasitica* (1) 6 mg/L, (2) 8 mg/L, (3) 10 mg/L on the 6*th* hour

TLC profiles of *G. walla* cell suspension under biological elicitation indicated a similarity to that of chemical elicitation with prominent light blue and maroon spots which, however, differed from spots observed in *G. walla* calli under biological elicitation (Fig. 11).

**Fig. 12:**
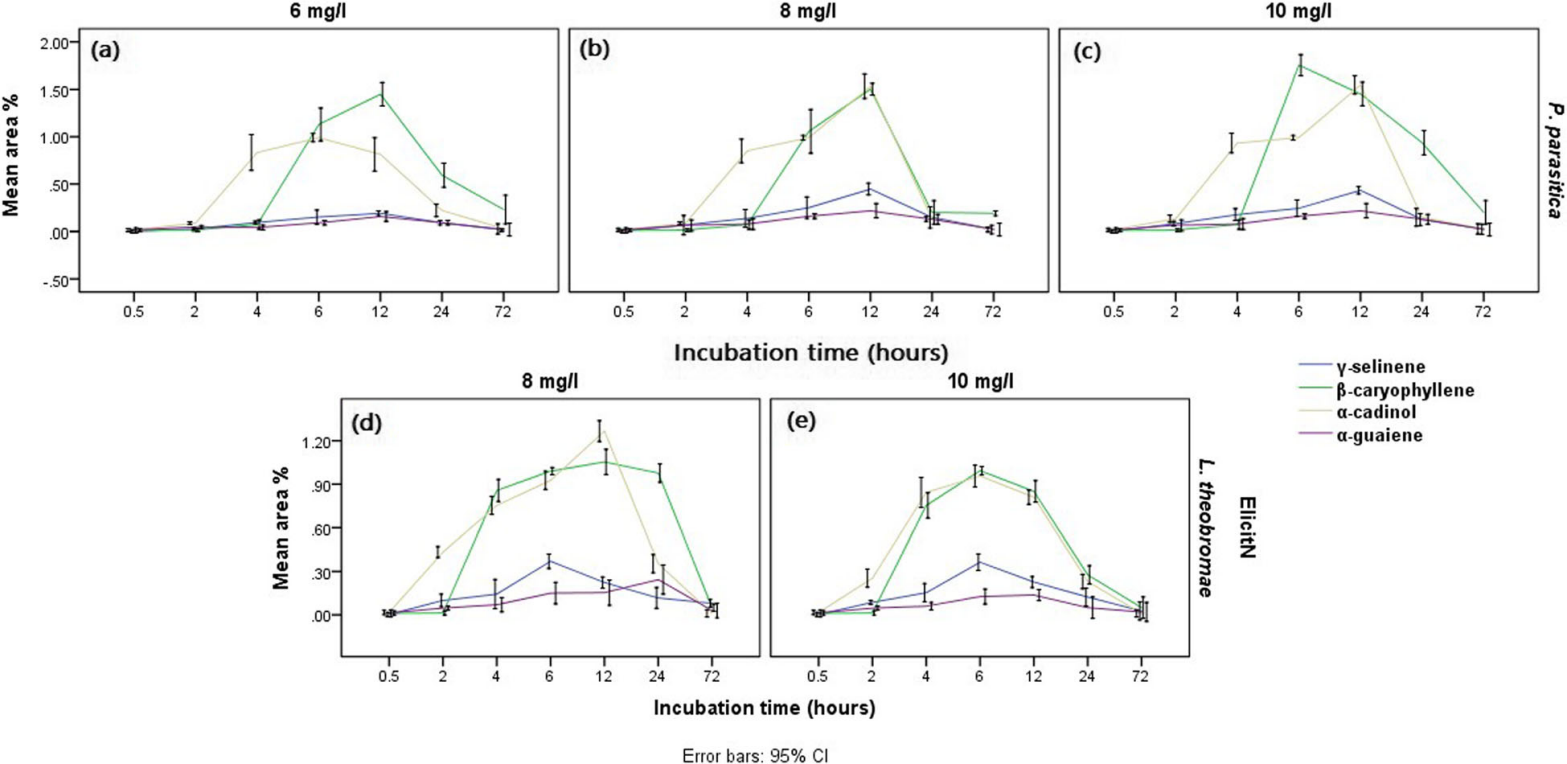
Mean area percentages of sesquiterpenes produced in *G. walla* cell suspension under (**a**) 6 mg/l, (**b**) 8 mg/l, (**c**) 10 mg/l *P. parasitica* and (**d**) 8 mg/l, (**e**) 10 mg/l *L. theobromae*

Graphical presentation of biological elicitation of *G. walla* cell suspensions are shown in Fig. 12. According to the figure, 6 mg/L, 8 mg/L and 10 mg/L of *Phaeocremonium parasitica* (Fig. 12 a, b and c) and 8 mg/L and 10 mg/L of *Lasidiplodia theobromae* (Fig. 12 d and e) on the 6th and 12th hour of incubation periods were more effective in the occurrence of sesquiterpenes. Comparatively, 8 mg/L and 10 mg/L *Phaeocremonium parasitica* resulted in a similar effect on *G. walla* cell suspension (Table 4). Conversely, higher area percentages of sesquiterpenes were obtained for 8 mg/L *Lasidiplodia theobromae* on the 6th (γ-selinene — 0.37 % and β-charyophllene — 0.99%), 12th (α-cadinol — 1.26%) and 24th hour (α-guaiene — 0.24 %). Similar to chemical elicitation of cell suspension culture, the area percentage of β-charyophllene and α-cadinol were higher; meanwhile, minimum area percentages of γ-selinene and γ-guaiene were obtained with biological elicitation of *G. walla* cell suspension.

**Table 4:** Effect of biological elicitors on *Gyrinops walla* cell suspension

Type of elicitation	Concentration (mg/L)	Incubation period (hours)	Area percentage of			
			γ-Selinene	β-Caryophyllene	α-Cardinol	α-Guaiene
Control	0	0	0.01 (0.005)^hi^	0.01 (0.001)^h^	0.01 (0.005)^i^	0.01 (0.005)^g^
PP	6.0	0.5	0.01 (0.005)^hi^	0.01 (0.001)^h^	0.01 (0.005)^i^	0.01 (0.005)^g^
PP	6.0	2	0.02 (0.005)^hi^	0.01 (0.005)^h^	0.08 (0.005)^i^	0.04 (0.001)^gf^
PP	6.0	4	0.09 (0.005)^gf^	0.07 (0.02)^h^	0.83 (0.07)^d^	0.04 (0.01)^gf^
PP	6.0	6	0.15 (0.03)^ed^	1.13 (0.07)^e^	**0.99 (0.01)**^**c**^	0.09 (0.01)^d^
PP	6.0	12	**0.19 (0.01)**^**d**^	**1.45 (0.05)**^**c**^	0.81 (0.07)^de^	**0.15 (0.02)**^**cb**^
PP	6.0	24	0.09 (0.01)^gf^	0.59 (0.05)^e^	0.24 (0.02)^f^	0.09 (0.01)^d^
PP	6.0	72	0.01 (0.005)^hi^	0.23 (0.06)^f^	0.02 (0.002)^i^	0.02 (0.001)^g^
PP	8.0	0.5	0.01 (0.005)^i^	0.01 (0.001)^h^	0.01 (0.005)^i^	0.01 (0.005)^g^
PP	8.0	2	0.06 (0.04)^hgi^	0.01 (0.005)^h^	0.08 (0.005)^i^	0.06 (0.02)^edf^
PP	8.0	4	0.13 (0.03)^edf^	0.07 (0.02)^h^	0.85 (0.05)^d^	0.07 (0.02)^ed^
PP	8.0	6	0.25 (0.04)^c^	1.05 (0.09)^b^	0.99 (0.01)^c^	0.16 (0.01)^b^
PP	8.0	12	**0.44 (0.02)**^**a**^	**1.50 (0.02)**^**b**^	**1.53 (0.05)**^**a**^	**0.21 (0.03)**^**a**^
PP	8.0	24	0.14 (0.04)^edf^	0.20 (0.005)^f^	0.14 (0.01)^h^	0.12 (0.02)^c^
PP	8.0	72	0.02 (0.001)^i^	0.19 (0.02)^f^	0.02 (0.01)^i^	0.02 (0.002)^g^
PP	10.0	0.5	0.003 (0.001)^i^	0.01 (0.001)^h^	0.01 (0.005)^i^	0.01 (0.002)^g^
PP	10.0	2	0.08 (0.01)^hgf^	0.01 (0.005)^h^	0.13 (0.01)^h^	0.06 (0.02)^edf^
PP	10.0	4	0.17 (0.02)^d^	0.07 (0.02)^h^	0.93 (0.04)^c^	0.07 (0.02)^ed^
PP	10.0	6	0.24 (0.03)^c^	**1.75 (0.04)**^**a**^	0.99 (0.01)^c^	0.16 (0.01)^b^
PP	10.0	12	**0.43 (0.01)**^**ab**^	1.45 (0.05)^d^	**1.54 (0.03)**^**a**^	**0.21 (0.03)**^**a**^
PP	10.0	24	0.12 (0.02)^edf^	0.93 (0.05)^d^	0.14 (0.03)^h^	0.12 (0.02)^c^
PP	10.0	72	0.02 (0.002)^i^	0.20 (0.05)^f^	0.02 (0.001)^i^	0.02 (0.004)^g^
LT	8.0	0.5	0.01 (0.005)^i^	0.01 (0.003)^h^	0.01 (0.005)^i^	0.01 (0.004)^g^
LT	8.0	2	0.10 (0.01)^egf^	0.01 (0.005)^h^	0.43 (0.01)^f^	0.04 (0.005)^ef^
LT	8.0	4	0.14 (0.04)^edf^	0.85 (0.03)^f^	0.75 (0.02)^fh^	0.07 (0.02)^ed^
LT	8.0	6	**0.37 (0.02)**^**b**^	**0.99 (0.01)**^**d**^	0.92 (0.02)^c^	0.15 (0.03)^cb^
LT	8.0	12	0.22 (0.01)^c^	1.05 (0.03)^e^	**1.26 (0.02)**^**b**^	0.15 (0.03)^cb^
LT	8.0	24	0.11 (0.02)^edf^	0.97 (0.02)^d^	0.35 (0.02)^f^	**0.24 (0.04)**^**a**^
LT	8.0	72	0.08 (0.01)^hgf^	0.45 (0.05)^de^	0.01 (0.001)^i^	0.03 (0.005)^g^
LT	10.0	0.5	0.01 (0.005)^i^	0.01 (0.001)^h^	0.01 (0.004)^i^	0.01 (0.005)^g^
LT	10.0	2	0.08 (0.005)^hgf^	0.01 (0.005)^h^	0.25 (0.02)^h^	0.04 (0.001)^ef^
LT	10.0	4	0.15 (0.02)^ed^	0.75 (0.03)^e^	0.84 (0.04)^cd^	0.06 (0.01)^edf^
LT	10.0	6	**0.36 (0.02)**^**b**^	**0.99 (0.01)**^**d**^	**0.95 (0.03)**^**c**^	0.12 (0.02)^c^
LT	10.0	12	0.22 (0.01)^c^	0.85 (0.03)^gf^	0.81 (0.02)^d^	**0.13 (0.01)**^**cb**^
LT	10.0	24	0.12 (0.02)^edf^	0.27 (0.02)^fg^	0.23 (0.02)^h^	0.05 (0.003)^ed^
LT	10.0	72	0.02 (0.002)^i^	0.05 (0.03)^h^	0.01 (0.003)^i^	0.02 (0.005)^g^

*PP*, *Phaeocremonium parasitica*; TV, *Trichoderma viride*; and *LT*, *Lasidiplodia theobromae.* The mean values are followed by standard deviation within parentheses. The same letter along the columns indicates no statistically significant difference at p ≤ 0.05

**Fig. 13:**
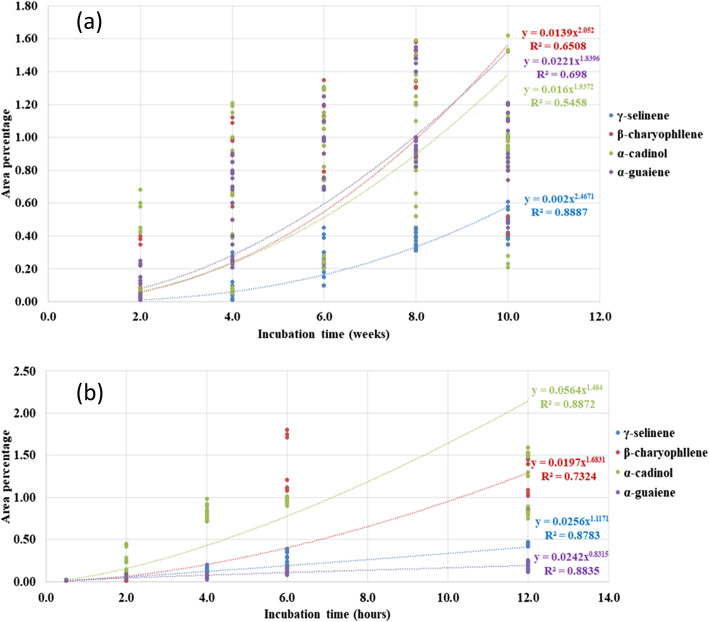
Trends of area percentages of biologically elicited (**a**) *G. walla* calli and (**b**) cell suspension. *The graphs include the data* up to the *maximum area percentage of sesquiterpenes*

According to Fig. 13, two distinct behavioural patterns of sesquiterpenes were observed for *G. walla* calli which consisted of β-caryophyllene, α-cadinol and α-guaiene (pattern 1) and γ-selinene (pattern 2). Trendlines of sesquiterpenes in pattern 1 overlapped with each other while pattern 2 was proceeded distantly from pattern 1. Comparatively, the area percentage of β-caryophyllene, α-cadinol and α-guaiene was higher than that of γ-selinene. Furthermore, trendlines of the sesquiterpenes showed an earlier production of α-guaiene which eventually dropped while β-caryophyllene was increased on the 10th week. On the contrary, three behavioural patterns of sesquiterpenes were observed in *G. walla* cell suspension elicited with biological elicitors which consisted of α-cadinol (pattern 1), β-caryophyllene (pattern 2) and, α-guaiene and γ-selinene (pattern 3). Similar to the trendlines observed in chemically elicited cell suspensions, the area percentage of α-cadinol was comparatively higher than that of biologically elicited calli. Trendlines of α-guaiene and γ-selinene performed similarly.

## Discussion

During the production of sesquiterpenes in artificially elicited callus and cell suspension cultures of *G. walla*, the peaks of different sesquiterpenes appeared and were rapidly attenuated, and the aroma simultaneously faded away in certain elicitation conditions after 8 weeks and 6 h, respectively. This implies that different pathways of sesquiterpenes of highly volatile to less volatile compounds are involved and the volatile compounds might escape from the system immediately. Further, during the elicitation, interaction among the sesquiterpenes and the feedback mechanisms involved in inhibition and activation of enzyme systems through gene expression could be one of the plausible explanations of the transient appearance of volatile compounds. Various mechanisms have been put forwarded to explain these observations such as degradation by endogenous enzymes [12] and diffusion of the aromatic compounds into the air which supports the findings of the present study. In addition, results of the study suggested that the viability of the calli and cell suspension are essential in the production of sesquiterpenes. However, in the production of chromone compounds, cell viability does not play a major role. Okudera and Ito [12] are of the opinion that chromone derivatives are produced by the degradation of cell wall constituents by endogenous enzymes during the process of cell death and sesquiterpenes are compounds whose biosynthesis is induced by signal transducers which happens when cells are viable. This finding supports the observations of the present study regarding the appearance of sesquiterpenes when calli/cell suspensions are viable. Moreover, the findings of the present study indicate that the maximum incubation period for *G. walla* calli and cell suspension are 8 or 10 weeks and 12 h, respectively. In the present study, sesquiterpenes α-cadinol, β-caryophyllene, α-guaiene and γ-selinene, which are identical to that of the wood, were obtained from the three elicitation methods. Moreover, by observing Figs. 6 and 13, it can be concluded that different elicitation methods yield sesquiterpenes in different compositions. All these findings imply the potential of artificially eliciting calli and cell suspensions of *G. walla* to induce synthesis of sesquiterpenes.

*G. walla* callus and cell suspension cultures treated with SA and MJ showed an enhancement of sesquiterpene content. The addition of SA and MJ yielded higher area percentages of α-cadinol and β-caryophyllene, and lower area percentages of α-guaiene and γ-selinene were observed in both *G. walla* calli and cell suspension. SA and MJ are well-known as signal transducers in response to wounding and pathogens [23]. Therefore, the occurrence of sesquiterpenes and associations between these sesquitepenes were supported by the fact that SA and MJ play important roles in plant defence, in which a variety of defence genes are exposed through “cross-talking” of these signal transducers [24, 25]. Several studies on *Aquilaria* cell cultures have shown that MJ and SA elicitation enhance the production of sesquiterpenes. For instance, Kumeta and Ito, [26] showed that the production of sesquiterpene compounds, α-humulene and guaiene derivatives, was induced by externally added MJ in *Aquilaria* cell suspensions. In their study, humulene and guaiene have different skeletal types, and their relative amounts in the suspension cells changed during the course of the culture period, which may mean that at least two different types of sesquiterpene cyclases were induced with different kinetics in *Aquilaria* suspension cells by MJ stimulation. The addition of MJ and SA to the callus and cell suspension cultures resulted in maximum sesquiterpene production, which indicated the potential of abiotic elicitors SA and MJ for the enhancement of sesquiterpene biosynthesis in callus and cell suspension cultures of *G. walla*.

The carbohydrate homogenates of *Phaeocremonium parasitica*, *Trichoderma viride* and *Lasidiplodia theobromae* fungal strains with their higher concentrations were effective in the production of sesquiterpenes in callus and cell suspension cultures of *G. walla*. Particularly, in *G. walla* calli, three sesquiterpenes, except γ-selinene, occurred in similar quantities indicating the productivity and efficiency of biological elicitation to *G. walla* calli compared to other elicitation methods. In order to achieve the maximum induction of sesquiterpene synthesis, both the dose of the elicitor and the age of culture to be elicited needed to be optimized. These findings are supported by similar studies conducted on several other metabolite and plant cell systems, e.g., shikonin production by *Lithospermum erythrorhizon* [27], shikonin by *Arnebia euchroma* [28], and taxoids by *Taxas cuspidate* [29]. The requirement of optimal dose suggests that at elicitor doses smaller than the optimum, the elicitor-binding sites in the cells were still not fully utilized for activating the secondary metabolite synthesis, while excessive doses caused a deleterious effect on the cells’ biosynthetic activity [30].

Various fungal elicitors, including cell wall fragments, polysaccharides, oligosaccharides and glycoproteins, have also been used in the induction of secondary metabolite production with many other plant cell species [27, 31]. However, it is still not well-understood how these elicitors mediated the secondary metabolite biosynthesis in plant cells. Some recent studies on one class of putative elicitors, oligoglucosides, suggested that elicitors first bind to certain proteins (the elicitor-binding proteins) in the cell membrane, which might function as signal transduction receptors to initiate a series of subsequent defence-related responses [32]. These responses may include the synthesis and incorporation of hydroxyproline-rich glycoproteins, cellulose callose and polymers, the production of phytoalexins, and the enhanced expression of genes encoding enzymes such as phenylalanine ammonia-lyase. However, the relevance of these responses to the enhancement of secondary metabolite synthesis remains to be discovered.

Calli and cell suspensions of several genera of family Thymelaeaceae have been subjected to biological elicitation for the production of sesquiterpenes, including *Aquilaria sinensis* induced by *Lasiodiplodia theobromae* [13] and *Aquilaria malaccensis* by *Trichoderma* [16]. However, as far as *G. walla* is concerned, there is scarcity in published records for biological elicitation of *G. walla* calli and cell suspension.

## Conclusion

The chemical, biological, elicitation of calli and cell suspension of *G. walla* proved the feasibility of production of four sesquiterpenes (γ-selinene, α-guaiene, β-caryophyllene and α-cadinol) under laboratory condition. Future perspective studies are required to affirm the feasibility of mass production of agarwood resinous substances using pilot-scale systems such as bioreactors with hairy root culture.

## Data Availability

N/A

## Abbreviations

BAP: 6-Benzylaminopurine
GC-MS: Gas chromatography-mass spectroscopy
LT: *Lasidiplodia theobromae*
MJ: Methyl jasmonate
MSM: Murashige and Skoog medium
NAA: 1-Naphthaleneacetic acid
PP: *Phaeocremonium parasitica*
PDA: Potato dextrose agar
SA: Salicylic acid
TTC: 2, 3, 5-Triphenyltetrazolium chloride
TLC: Thin-layer chromatography
TV: *Trichoderma viride*
UV: Ultraviolet

## References

[1] Herber BE, Metcalfe CR, Chalk L (1988). Anatomy of the dicotyledons. Systematic anatomy of the leaf and stem, with a brief history of the subject.

[2] Compton JGS, Zich FA (2002). *Gyrinops ledermannii* (Thymalaeaceae), being an agarwood producing species prompts call for further examination of taxonomic implications in the generic delimitation between *Aquilaria* and *Gyrinops*. Flora Malesiana Bull.

[3] Nobuchi T, Siripatanadilok S (1991). Preliminary observation of *Aquilaria crassna* wood associate with the formation of aloeswood. Bull Kyoto Univ Forests.

[4] Takemoto H, Ito M, Shiraki T, Yagura T, Honda G (2008). Sedative effects of vapor inhalation of agarwood oil and spikenard extract and identification of their active components. J Nat Med.

[5] www.dailynews.lk. Retrived July 7, 2019 from www.dailynews.lk/2018/06/28/local/155233/dubai-bound-passenger-nabbed-walla-patta?page=11.

[6] UNEP-WCMC (2019) CITES trade statistics derived from the CITES Trade Database. Retrieved 15/09/2019, from UNEP-World Conservation Monitoring Centre http://trade.cites.org/

[7] MOE (2012). The Notational Red List 2012 of Sri Lanka. Conservation status of the Fauna and Flora.

[8] Karuppusamy S (2009). A review on trends in production of secondary metabolites from higher plants by in vitro tissue, organ and cell cultures. J Med Plant Res.

[9] Lila KM (2005) Valuable secondary products from *in vitro* culture, Secondary Products *In Vitro*. CRC Press LLC

[10] Vijayasree N, Udayasri P, Aswani KY, Ravi BB, Phani KY, Vijay VM (2010). Advancements in the production of secondary metabolites. J Nat Prod.

[11] Qi SY, He ML, Lin LD, Zhang CH, Hu LJ, Zhang HZ (2005). Production of 2-(2-phenylethyl) chromones in cell suspension cultures of *Aquilaria sinensis*. Plant Cell Tissue Organ Cult.

[12] Okudera Y, Ito M (2009). Production of agarwood fragrant constituents in *Aquilaria* calli and cell suspension cultures. Plant Biotechnol.

[13] Zhang Z, Han XM, Wei JH, Xue J, Yang Y, Liang L, Gao ZH (2014). Compositions and antifungal activities of essential oils from agarwood of *Aquilaria sinensis* (Lour.) Gilg induced by *Lasiodiplodia theobromae* (Pat.) Griffon. & Maubl. J Braz Chem Soc.

[14] Crous PW, Gams W, Wingfield MJ, Van Wyk PS (1996). *Phaeoacremonium* gen. nov. associated with wilt and decline diseases of woody hosts and human infections. Mycologia.

[15] Sangareswari M, Parthiban KT, Kanna SU, Karthiba L, Saravanakumar D (2016). Fungal microbes associated with agarwood formation. Am J Plant Sci.

[16] Jayaraman S, Mohamed R (2015). Crude extract of *Trichoderma* elicits agarwood substances in cell suspensioculture of the tropical tree, Aquilaria malaccensis Lam. Turk J Agric For.

[17] Munasinghe SP, Somaratne S, Weerakoon SR, Ranasinghe C (2020). Prediction of chemical composition for callus production in *Gyrinops walla* Gaetner through machine learning. Inform Process Agric.

[18] Duncan DR, Widholm JM (1990) Measurements of viability suitable for plant tissue cultures. In: Plant cell and tissue culture. Humana Press, pp 29–37 10.1385/0-89603-161-6:29 21390590

[19] Mustafa NR, De Winter W, Van Iren F, Verpoorte R (2011) Initiation, growth and cryopreservation of plant cell suspension cultures. Nat Protoc 6(6):715–742. 10.1038/nprot.2010.144 10.1038/nprot.2010.144 21637194

[20] Ito M, Okimota KL, Yagura T, Honda G, Kiuchi F, Shimada Y (2005) Induction of sesquiterpenoid production by methyl jasmonate in Aquilaria sinensis cell suspension culture. Journal of Essentila oil Research 17(20):175-180

[21] Dubois M, Gilles K, Hamilton J, Rebers P, Smith F (1956). Colorimetric method based on phenol sulfuric acid. Anal Chem.

[22] Jain SC, Pancholi B, Jain R (2012). *In-vitro* callus propagation and secondary metabolite quantification in *Sericostoma pauciflorum*. Iran J Pharm Res.

[23] Shah J (2003). The salicylic acid loop in plant defense. Curr Opin Plant Biol.

[24] Felton GW, Korth KL (2000). Trade-offs between pathogen and herbivore resistance. Curr Opin Plant Biol.

[25] Reymond P, Farmer EE (1998). Jasmonate and salicylate as global signals for defense gene expression. Curr Opin Plant Biol.

[26] Kumeta Y, Ito M (2010). Characterization of δ-guaiene synthases from cultured cells of *Aquilaria*, responsible for the formation of the sesquiterpenes in agarwood. Plant Physiol.

[27] Chang HN, Sim SJ (1994). Increasing secondary metabolite production in plant cell cultures with fungal elicitors. Biotechnological applications of plant cultures.

[28] Fu XQ, Lu DW (1999). Stimulation of shikonin production by combined fungal elicitation and *in situ* extraction in suspension cultures of *Arnebia euchroma*. Enzym Microb Technol.

[29] Ketchum RE, Rithner CD, Qiu D, Kim YS, Williams RM, Croteau RB (2003). Taxus metabolomics: methyl jasmonate preferentially induces production of taxoids oxygenated at C-13 in Taxus x media cell cultures. Phytochemistry.

[30] Wang C, Wu J, Mei X (2001). Enhancement of taxol production and excretion in Taxus chinensis cell culture by fungal elicitation and medium renewal. Appl Microbiol Biotechnol.

[31] DiCosmo F, Misawa M (1985). Eliciting secondary metabolism in plant cell cultures. Trends Biotechnol.

[32] Ebel J, Mithöfer A (1998). Early events in the elicitation of plant defence. Planta.

